# The Use of Marble Dust, Bagasse Ash, and Paddy Straw to Improve the Water Absorption and Linear Shrinkage of Unfired Soil Block for Structure Applications

**DOI:** 10.3390/ma15217786

**Published:** 2022-11-04

**Authors:** Tarun Sharma, Sandeep Singh, Shubham Sharma, Prashant Sharma, Anita Gehlot, Anand Kumar Shukla, Sayed M. Eldin

**Affiliations:** 1 Department of Civil Engineering, Chandigarh University, Mohali 140413, Punjab, India; 2 Department of Civil Engineering, University Center for Research and Development, Chandigarh University, Mohali 140413, Punjab, India; 3 Mechanical Engineering Department, University Center for Research and Development, Chandigarh University, Mohali 140413, Punjab, India; 4 School of Mechanical and Automotive Engineering, Qingdao University of Technology, Qingdao 266520, China; 5 Department of Civil Engineering, GLA University, Mathura 281406, Uttar Pradesh, India; 6 Uttaranchal Institute of Technology, Uttaranchal University, Dehradun 248007, Uttarakhand, India; 7 Department of Project Management, Universidad Internacional Iberoamericana, Campeche 24560, Mexico; 8 Chandigarh School of Business, Chandigarh Group of Colleges, Jhanjheri, Mohali 140308, Punjab, India; 9 Centre for Research, Faculty of Engineering, Future University in Egypt, New Cairo 11835, Egypt

**Keywords:** linear shrinkage, water absorption, paddy straw, marble dust, bagasse ash, compacted stabilized soil block

## Abstract

Unfired admixed soil blocks are made up of soil plus stabilizers such as binders, fibers, or a combination of both. Soil is abundant on Earth, and it has been used to provide shelter to millions of people. The manufacturing and usage of cement and cement blocks raise several environmental and economic challenges. Due to disposal issues, agricultural and industrial waste is currently the biggest hazard to the environment and humanity in the world. Consequently, environmental degradation brought on by agricultural waste harms the ecology. As a result, researchers are attempting to develop an alternative to cement blocks, and various tests on unfired admixed soil blocks have been done. This investigation uses agricultural waste (i.e., paddy straw fiber and sugarcane bagasse ash) and industrial waste (i.e., marble dust) in manufacturing unfired admixed soil blocks. Under this investigation, the applicability of unfired soil blocks admixed with marble dust, paddy straw fiber, and bagasse ash was studied. The marble dust level ranged from 25% to 35%, bagasse ash content ranged from 7.5% to 12.5%, and the content of paddy straw fiber ranged from 0.8% to 1.2% by soil dry weight. Various tests were conducted on the 81 mix designs of the prepared unfired admixed soil blocks to find out the physical properties of the block followed by modeling and optimization. The findings demonstrate that the suggested method is a superior alternative to burned bricks for improving the physical properties of admixed soil blocks without firing.

## 1. Introduction

Compacted stabilized adobe blocks are construction units made by adding the right amount of water to the right kind of soil to achieve maximum density and compressing it with the right block-forming machine [1,2]. Hand-operated or mechanically operated block-making equipment is available. As compared to burnt earth bricks, it is more environmentally friendly when used to make compacted stabilized soil blocks. Compacted soil stabilized blocks differ from fired earth bricks in that they do not require the use of a brick kiln, which produces a lot of pollution. As a result, researchers are attempting to develop an alternative to cement blocks, and various tests on unfired admixed soil blocks have been done. The soil mixture is deposited in the press chamber for block production. When the soil–cement mixture is subjected to force, the material is compressed, eliminating voids while increasing density. The higher the density that may be attained, the lower the porosity of the soil [3]. The proctor test may be used to assess the proper moisture level as well as the relationship between the maximum dry density and the moisture content [4]. The different binders and fibers are used for the manufacturing of unfired admixed soil blocks [5,6,7,8]. Bitumen emulsion, cement, grit [9], sugarcane bagasse ash, limestone waste, lime, calcium silicate [10], limestone residues [11], granite waste, demolition residue [12], kaolin, rice husk ash, Bacillus pasteurii KCTC 3558 [13], construction debris, fly ash, green mussel shell powder [14], and effective microorganisms (EMs) are some of the binders used. Natural and synthetic fibers have been employed in various research, with coconut fiber being the most commonly used fiber. Gutiérrez-Orrego et al. found that the inclusion of sisal fiber resulted in a modest drop in density [15]. Except in the case of [16], all investigations have revealed that water absorption levels are within allowed limits, i.e., <20% [17,18,19,20]. Except for cement, fibers were shown to be more successful than binders in lowering soil block water absorption (WA). According to [7,15,17], the reduction in WA could be related to the incorporation of fibers, which lowers shrinkage cracks caused by the drying. This study aimed to look at the impact of diverse wastes, i.e., marble dust (MD), paddy straw fiber (PSF), and bagasse ash (BA) on the physical attributes of unfired admixed adobe blocks, followed by its modeling and optimization. The marble dust is composed of a sufficient quantity of CaO, and bagasse ash is composed mainly of SiO_2_, as shown in the section of materials and methods, which results in providing pozzolanic action on treatment with water. The addition of paddy straw fiber in conjunction with marble dust and bagasse ash results in the reduction of water absorption and linear shrinkage of the unfired admixed soil block. This study helps in providing one of the alternative solutions to the disposal problem of bagasse ash, marble dust, and paddy straw fiber and hence reducing environmental pollution.

## 2. Materials and Methods

### 2.1. Materials

The soil for this investigation was collected in Gharuan, Kharar (Punjab), India. Table 1 shows the engineering characteristics of the soil sample. It was found to be of CI (intermediate plasticity clay) type of soil. The PSF was obtained from Gharuan agricultural land near Chandigarh University. Paddy straw fibers (PSF) were chopped into required lengths of 75 mm, 100 mm, and 125 mm. Paddy straw with an average width of 2 mm was employed in the study.

Marble dust was bought from a business in Mohali, Punjab called Ram Lakhan Marble House. Table 2 demonstrates the XRF chemical composition of marble dust powder. In Table 2, it is shown that marble dust is mainly composed of calcium oxide (CaO). The specific gravity of the marble dust used for the study was 2.71. The marble dust size distribution by mechanical sieving showed that it contains 61% sand (0.05 mm to 2 mm size), 14% clay (less than 0.002 mm size), and 23% silt (0.002 mm to 0.05 mm size), which suggests that it belongs in the sand category.

Doaba Cooperative Sugar Mills Ltd. in Nawanshahr, Punjab provided bagasse ash for this study. Table 3 shows the chemical characteristics of bagasse ash (BA) as determined by an X-ray fluorescence test. In Table 3, it is shown that BA is mainly composed of silicon oxide (SiO_2_) and lower content of calcium oxide (CaO), potassium oxide (K_2_O), and magnesium oxide (MgO). The specific gravity of the BA used for the study was 1.92. Bagasse ash’s particle size ranged from 0 to 100 mm, making it comparable to ordinary Portland cement.

### 2.2. Experiment Overview

To test the influence of admixtures on the characteristics of soil blocks, a design mix was created, as listed in Table 4. Marble dust levels ranged from 25% to 35%, bagasse ash content ranged from 7.5% to 12.5%, and the content of paddy straw fiber ranged from 0.8% to 1.2% by soil dry weight. The length of paddy straw fiber was also varied: 75 mm, 100 mm, and 125 mm. Based on literature analysis, these percentages revealed that adding these components enhanced the characteristics of compacted soil blocks. It has been demonstrated that when 30% of soil is replaced with marble dust, compressive strength is maximized [11]; however no previous study for marble dust addition of 25% and 35% in soil blocks has been identified. For bagasse ash, 10% soil replacement had the greatest results [21,22]. There was no research on the use of paddy straw fiber, but based on research on other natural fibers, 1% fiber content of length 100 mm produced superior results [6,8], and thus 0.8%, 1%, and 1.2% fiber content were added to different mixtures, respectively. Each mix was tested three times before the average was utilized to determine the outcome. A total of 243 specimens were created from 81 combinations.

### 2.3. Specimen Preparation

**Figure 1:**
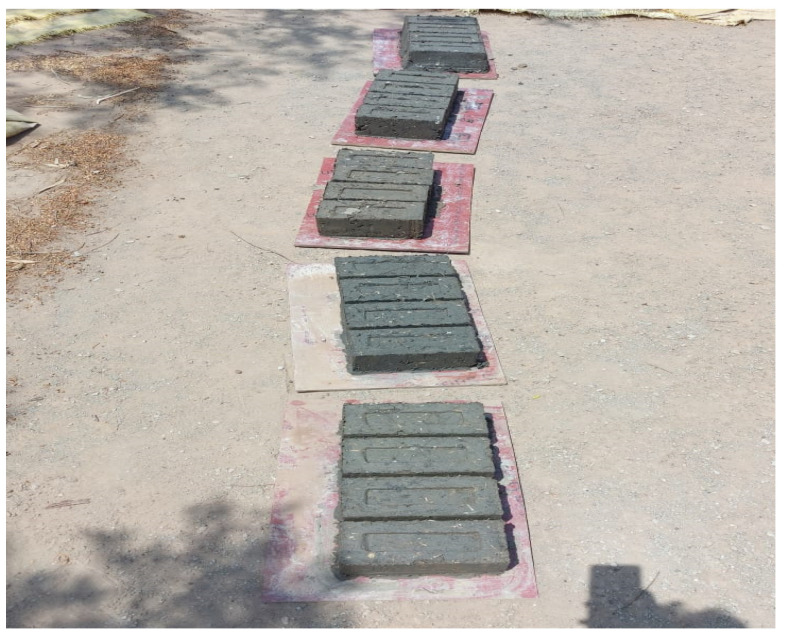
Admixed adobe blocks manufactured from the machine.

The soil was prepared according to BIS [23]. Paddy straw fibers were chopped into 75 mm, 100 mm, and 125 mm lengths. A 300-micron IS sieve was used to sift the marble dust and sugarcane bagasse ash. All these materials were mixed in a trolley using a step-by-step process. Then, for the specific experimental design mix, according to the OMC (optimum moisture content) acquired from the proctor compaction test, 50% of the water was added and the remaining water was added for thorough mixing. A block size of 230 mm × 100 mm × 100 mm was employed in this investigation. These solid blocks were manufactured with the help of a machine that generated 4 unfired admixed adobe blocks per pressing as shown in Figure 1.

The curing of the blocks was performed with the help of a jute bag. The set of blocks was cured with the help of sprinkling water and then covered with a jute bag till the next cycle of curing. This process of curing was repeated for 28 days. The final unfired admixed adobe block was in its finished form after 28 days of curing and was used for further testing of physical parameters. When measuring the physical qualities of soil blocks, the water absorption test is highly significant. According to IS 3495 (Part 2):1992, [24] the admixed adobe blocks were dried at 105 °C with the help of an oven until the mass is constant. After allowing the sample to cool to room temperature, the initial weight (W1) was recorded. The sample was then immersed in water for one day and kept at room temperature (27 plus/minus 2 °C). The sample was then removed, cleaned with a moist towel, and weighed (W_2_) after 3 min. The difference between the two weights (D = W_2_ − W_1_), reported as a percentage of dry weight, was used to calculate water absorption (in percentage). The water absorption (in percentage) of the admixed adobe block was determined using Equation (1):

(1) $$\frac{D}{W_{1}}\times 100$$

The linear shrinkage (LS) was calculated by comparing the length before and after 24 h of drying at temperatures ranging from 50 °C to 60 °C. According to the methodology adopted by [7], the initial length (L_1_) of admixed adobe block was measured and then kept in the oven for 24 h at 50 °C to 60 °C for drying. The sample was then removed from the oven, brought to room temperature and the final length (L_2_) after drying was recorded. The difference in length (C = L_2_ − L_1_) computed in percentage was used to calculate linear shrinkage. The linear shrinkage (in percentage) of the admixed adobe block was determined using Equation (2):

(2) $$\frac{C}{L_{1}}\times 100$$

## 3. Results and Discussion

### 3.1. Water Absorption of Unfired Admixed Soil Blocks

**Figure 2:**
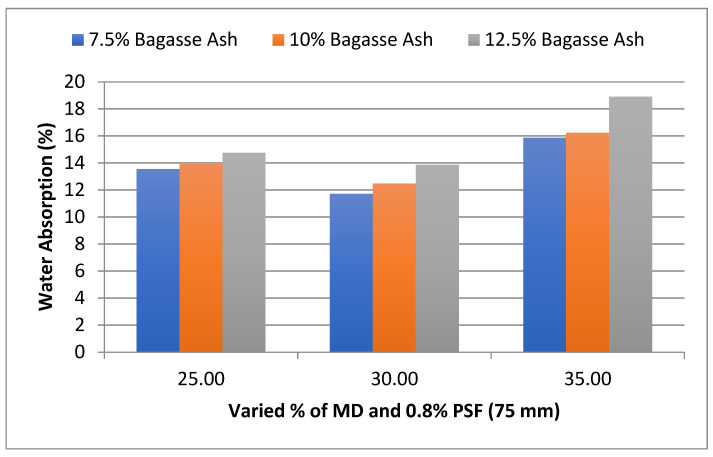
Effect of MD and BA on water absorption of block reinforced with 0.8% PS fiber (75 mm).

In this section, the influence of MD and BA on the water absorption (WA) of adobe blocks admixed with PSF of various contents and lengths was studied. As shown in Figure 2, it was found that water absorption of adobe block admixed with 0.8% PSF of length 75 mm showed firstly a decrement with an increase in marble dust and then gradual increase towards 35% marble dust at constant bagasse ash content. At 25% MD and 7.5% BA, the water absorption of the admixed adobe block reinforced with paddy straw fiber was 13.54%, decreased to 11.72% with 7.5% bagasse ash and 30% marble dust, and then increased to 15.87% with 7.5% bagasse ash and 35% marble dust.

However, it was observed from the experimental results that water absorption showed an increment with an increase in bagasse ash at constant marble dust content. Increased SBA concentration further increases water absorption according to Greepala and Parichartpreecha [25] and Singh and Kumar [19]. The water absorption values for all residual incorporations, however, remained below the 20% limitations specified by the IS: 3495 (Part 2) 1992 standard.

**Figure 3:**
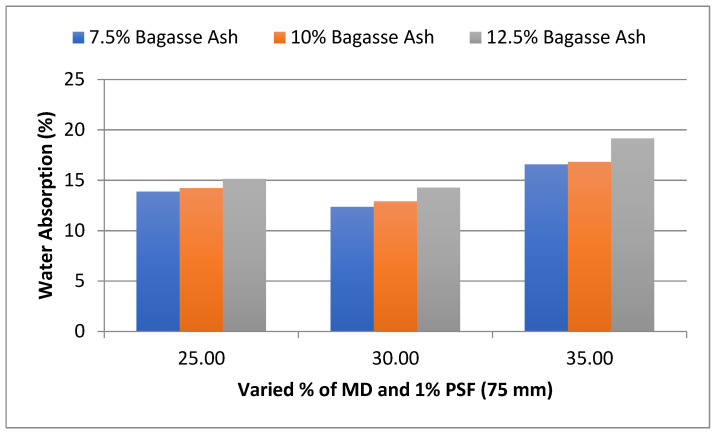
Effect of MD and BA on water absorption of block reinforced with 1% PS fiber (75 mm).

**Figure 4:**
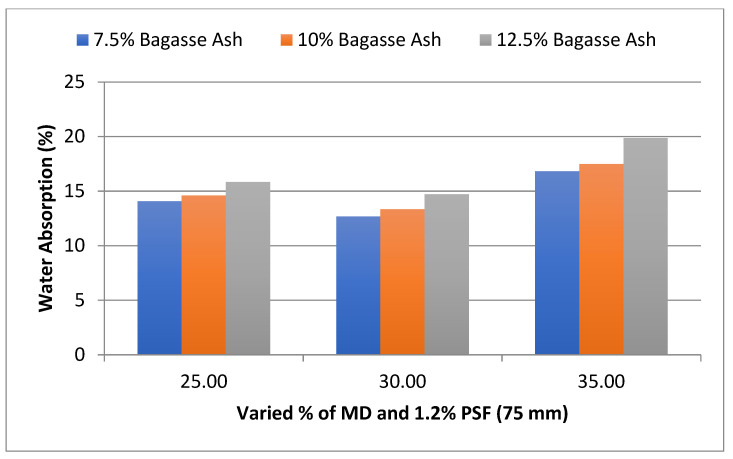
Effect of MD and BA on water absorption of block reinforced with 1.2% PS fiber (75 mm).

The influence of MD and BA on the water absorption of adobe block admixed with 1% and 1.2% paddy straw fiber of length 75 mm was also studied, as shown in Figure 3 and Figure 4, respectively. It was found that WA showed a similar trend to these PSF contents too.

**Figure 5:**
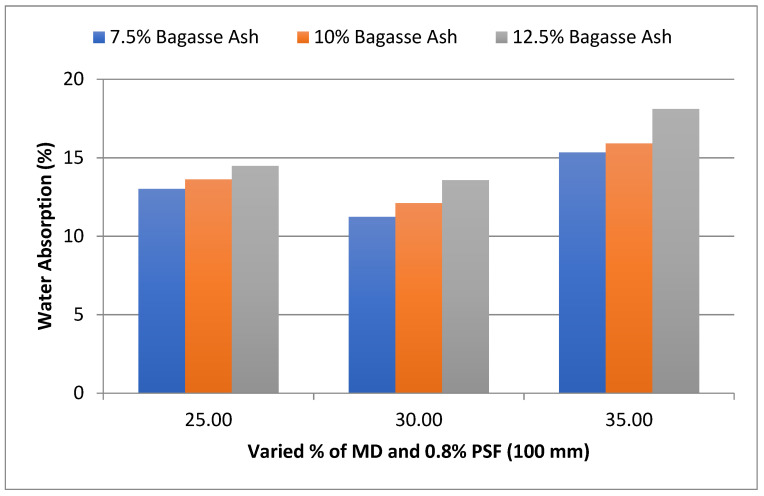
Effect of MD and BA on water absorption of block reinforced with 0.8% PS fiber (100 mm).

For 100 mm and 125 mm PSF, a similar effect of MD and BD on Water absorption was observed. As shown in Figure 5, at 25% MD and 7.5% BA, the water absorption of the admixed adobe block reinforced with 0.8%, 100 mm paddy straw fiber was 13.01%, decreased to 11.23% with 7.5% bagasse ash and 30% marble dust, and then increased to 15.34% with 7.5% bagasse ash and 35% marble dust.

**Figure 6:**
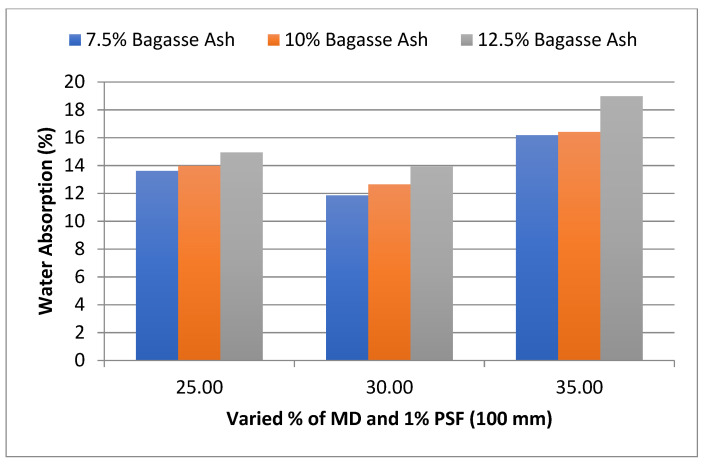
Effect of MD and BA on water absorption of block reinforced with 1% PS fiber (100 mm).

**Figure 7:**
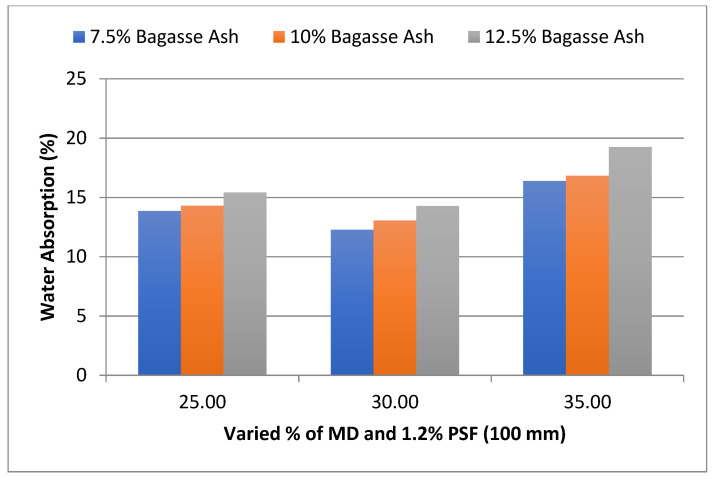
Effect of MD and BA on water absorption of block reinforced with 1.2% PS fiber (100 mm).

The influence of MD and BA on the water absorption of adobe block admixed with 1% and 1.2% paddy straw fiber of length 100 mm was also studied, as shown in Figure 6 and Figure 7. It was found that WA showed a similar trend to these PSF contents too. It was also observed from the graphs that WA tends to increase with an increase in PSF content at constant MD, BA, and PSF length.

**Figure 8:**
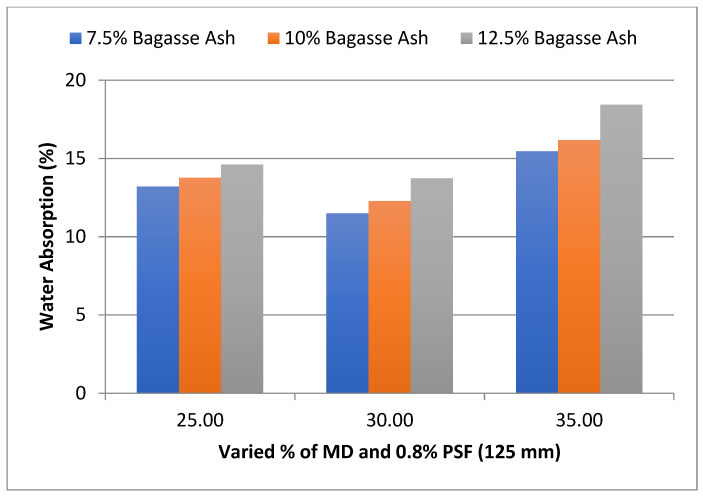
Effect of MD and BA on water absorption of block reinforced with 0.8% PS fiber (125 mm).

As shown in Figure 8, at 25% MD and 7.5% BA, the water absorption of the admixed adobe block reinforced with 0.8%, 125 mm paddy straw fiber was 13.21%, decreased to 11.49% with 7.5% bagasse ash and 30% marble dust, and then increased to 15.46% with 7.5% bagasse ash and 35% marble dust.

**Figure 9:**
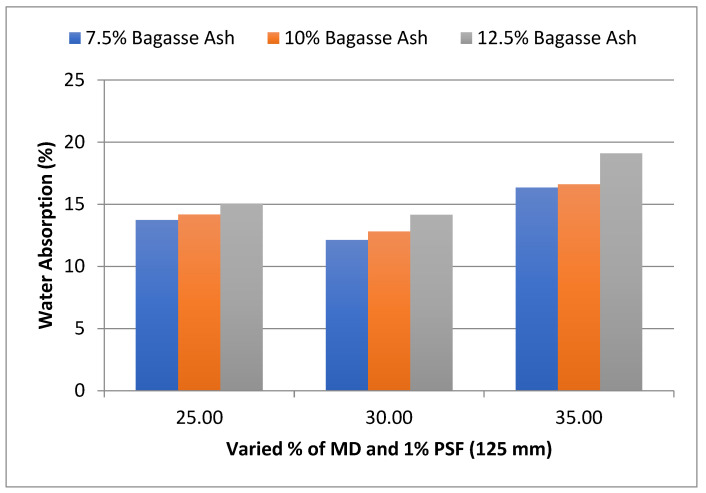
Effect of MD and BA on water absorption of block reinforced with 1% PS fiber (125 mm).

**Figure 10:**
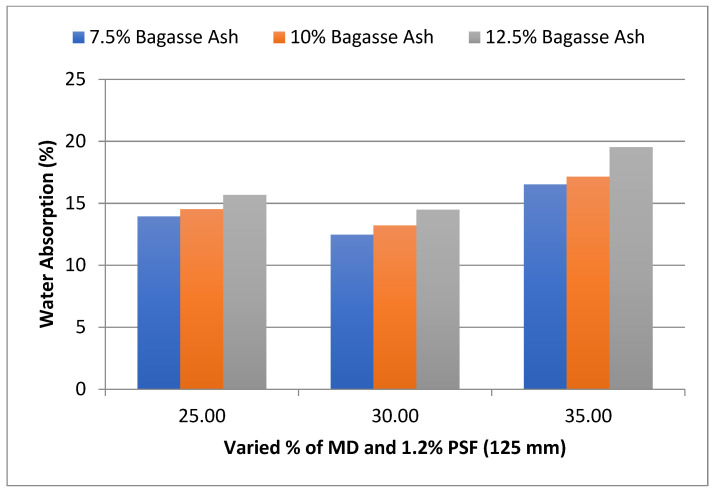
Effect of MD and BA on water absorption of block reinforced with 1.2% PS fiber (125 mm).

The influence of MD and BA on the water absorption of adobe block admixed with 1% and 1.2% paddy straw fiber of length 125 mm was also studied, as shown in Figure 9 and Figure 10, respectively. It was found that WA showed a similar trend with these PSF contents too. It was also observed from the graphs that WA tends to decrease with an increase in PSF length at constant MD, BA, and PSF content.

### 3.2. Linear Shrinkage of the Unfired Admixed Soil Block

**Figure 11:**
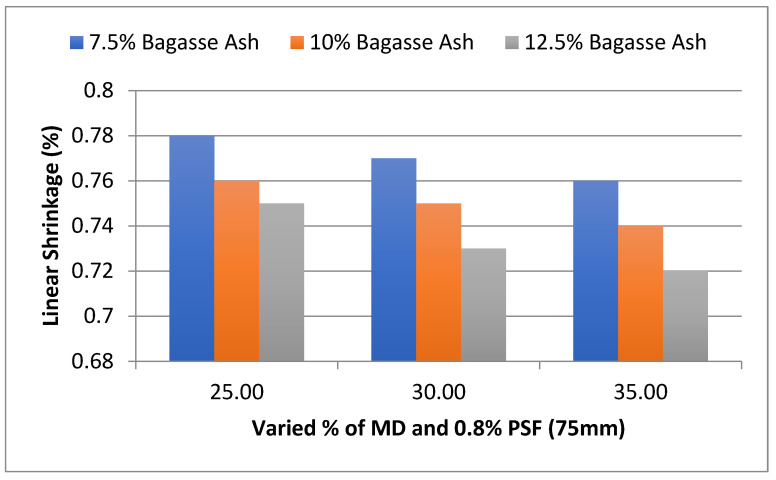
Effect of MD and BA on linear shrinkage of block reinforced with 0.8% PS fiber (75 mm).

The influence of MD and BA on the linear shrinkage (LS) of adobe blocks admixed with PSF of various contents and lengths was studied. As shown in Figure 11, it was found that linear shrinkage (%) showed a declining tendency with the rise in marble dust at constant bagasse ash content [26,27,28]. At 25% MD and 7.5% BA, the linear shrinkage of the adobe block reinforced with paddy straw fiber was 0.78%, which decreased to 0.76% with 7.5% bagasse ash and 35% marble dust. A similar trend was observed for 10% bagasse ash and 12.5% bagasse ash. A similar trend was observed with an increase in bagasse ash for constant marble dust content. At 25% MD and 7.5% BA, the linear shrinkage (%) of the adobe block reinforced with paddy straw fiber was 0.78%, which decreased to 0.75% with 12.5% bagasse ash and 25% marble dust. A similar trend was observed for 30% marble dust and 35% marble dust [17]. According to many codes of practice, the maximum allowable linear shrinkage is 3% [29,30,31], and in our study, the values of linear shrinkage were well within the permissible limits.

**Figure 12:**
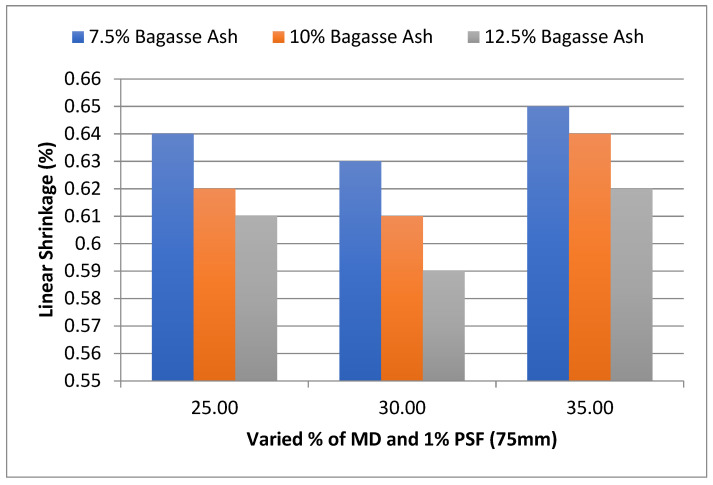
Effect of MD and BA on linear shrinkage of block reinforced with 1% PS fiber (75 mm).

The effect of MD and BA on the linear shrinkage of adobe block admixed with 1% paddy straw fiber of length 75 mm was also studied, as shown in Figure 12. It was found that linear had showed firstly a decrement with an increase in marble dust and then a gradual increase towards 35% marble dust at constant bagasse ash content. However, it was observed from the experimental results that linear shrinkage showed a decrement with an increase in bagasse ash at constant marble dust content [32,33,34].

**Figure 13:**
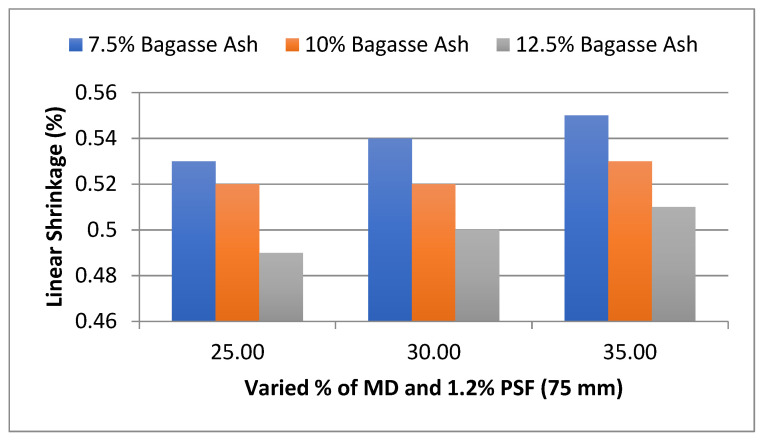
Effect of MD and BA on linear shrinkage of block reinforced with 1.2% PS fiber (75 mm).

The results also show that as the fiber content of the upgraded soil blocks increased, the linear shrinkage of the soil blocks reduced [7]. The inclusion of the fibers minimizes shrinkage by preventing the soil matrix from deforming due to friction [17,35,36]. Furthermore, a greater amount of fiber is more effective in preventing shrinkage cracks. In the management of linear shrinkage, fiber length is also a significant factor [8,37]. The effect of MD and BA on the linear shrinkage of adobe block admixed with 1.2% PSF of length 75 mm was studied, as shown in Figure 13. It was found that linear shrinkage showed an uptrend with an increase in marble dust at constant bagasse ash content. However, the opposite trend was observed with an increase in bagasse ash for constant marble dust content [36,37].

**Figure 14:**
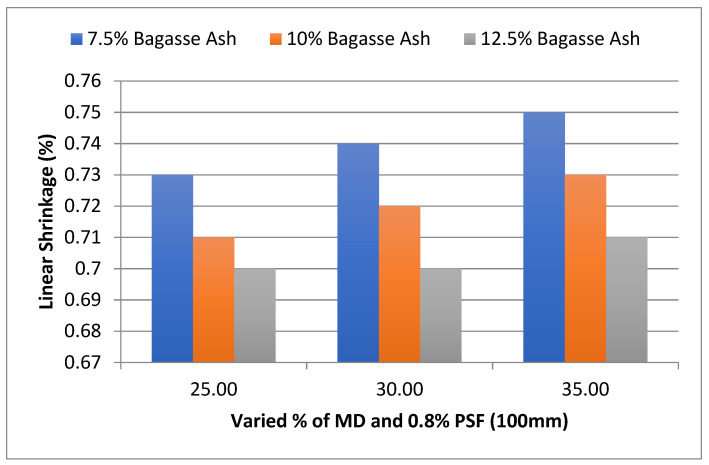
Effect of MD and BA on linear shrinkage of block reinforced with 0.8% PS fiber (100 mm).

For 100 mm and 125 mm PSF, a similar effect of MD and BD on linear shrinkage was observed. As shown in Figure 14, at 25% MD and 7.5% BA, the linear shrinkage of the admixed adobe block reinforced with 0.8%, 100 mm paddy straw fiber was 0.73%, which increased to 0.75% with 7.5% bagasse ash and 35% marble dust.

**Figure 15:**
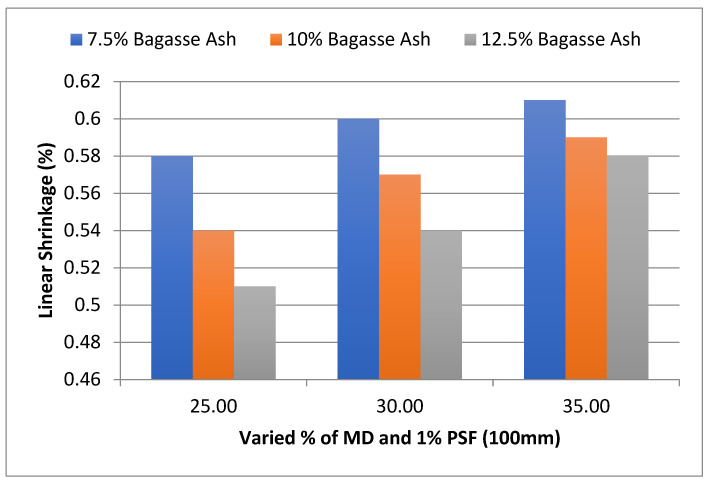
Effect of MD and BA on linear shrinkage of block reinforced with 1% PS fiber (100 mm).

**Figure 16:**
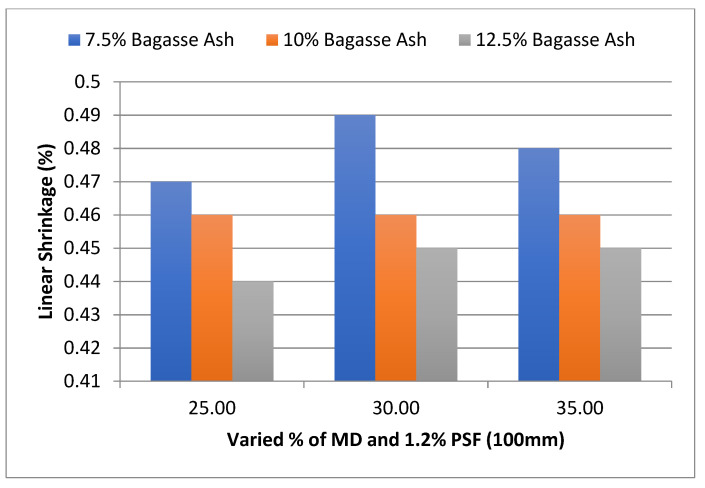
Effect of MD and BA on linear shrinkage of block reinforced with 1.2% PS fiber (100 mm).

In addition, the effect of MD and BA on the linear shrinkage of adobe block admixed with 1% and 1.2% paddy straw fiber of length 100 mm was studied, as shown in Figure 15 and Figure 16, respectively. It was found that linear shrinkage showed a similar trend to these PSF contents too.

**Figure 17:**
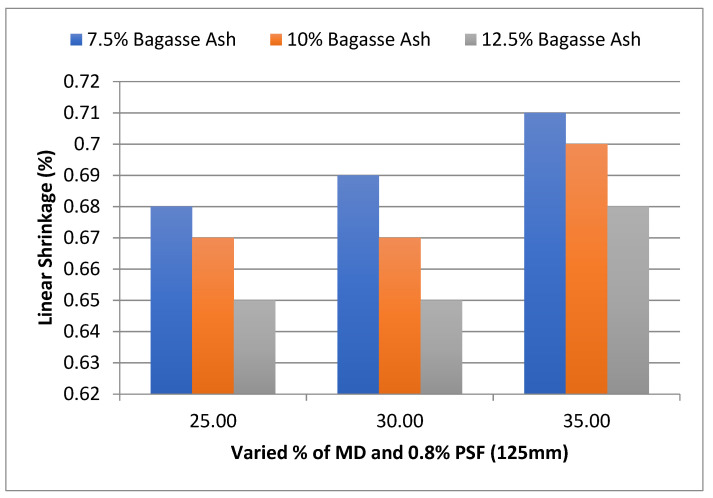
Effect of MD and BA on linear shrinkage of block reinforced with 0.8% PS fiber (125 mm).

As shown in Figure 17, at 25% MD and 7.5% BA, the linear shrinkage of the admixed adobe block reinforced with 0.8%, 125 mm paddy straw fiber was 0.68%, which increased to 0.71% with 7.5% bagasse ash and 35% marble dust.

**Figure 18:**
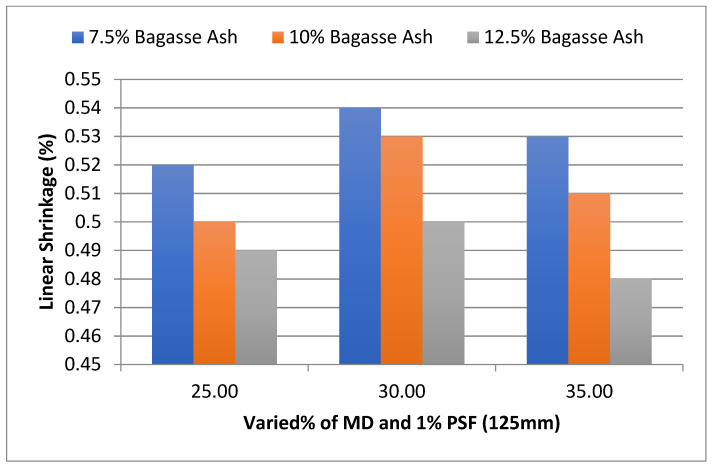
Effect of MD and BA on linear shrinkage of block reinforced with 1% PS fiber (125 mm).

**Figure 19:**
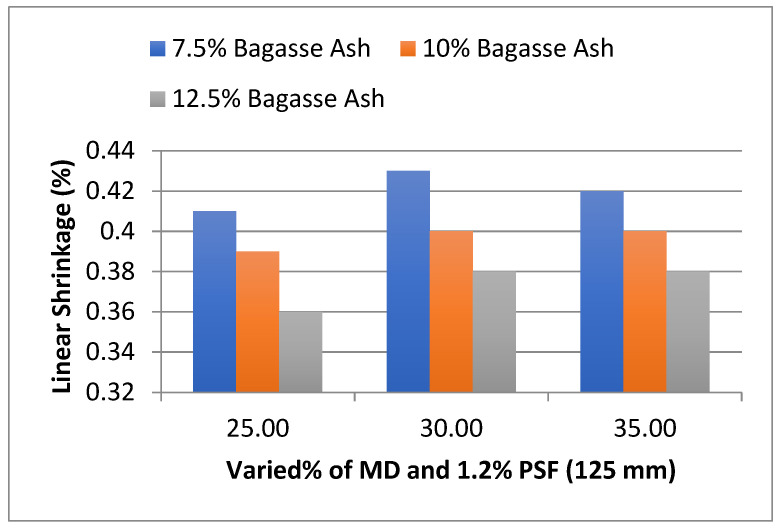
Effect of MD and BA on linear shrinkage of block reinforced with 1.2% PS fiber (125 mm).

In addition, the effect of MD and BA on the linear shrinkage of adobe block admixed with 1% and 1.2% paddy straw fiber of length 125 mm was studied, as shown in Figure 18 and Figure 19. It was found that linear shrinkage showed an increment with an increase in marble dust to 30% marble dust and then a gradual decrease towards 35% marble dust at constant bagasse ash content.

However, it was observed from the experimental results that linear shrinkage showed a decrement with an increase in bagasse ash at constant marble dust content. It was found that linear shrinkage showed a similar trend with 1.2% PSF content too.

### 3.3. Statistical Analysis

Minitab 17.1 software was utilized for regression analysis, and a fit regression model was used for the generation of model equations, which were further used for optimization.

#### 3.3.1. Model Equation: Water Absorption versus x1, x2, x3, x4

The association between parameters and water absorption (WA) was developed using regression analysis, and the result is shown in Equation (3), a model equation to determine WA of unfired soil block admixed with the varied proportion of PSF, MD, and BA:

WA = 120.08 − 0.0929 x1 + 3.42 x2 − 2.104 x3 − 6.781 x4 + 0.000466 x1*x1 − 1.57 x2*x2 + 0.08184 x3*x3 + 0.11275 x4*x4 + 0.00100 x1*x2 + 0.000231 x1*x3 − 0.000253 x1*x4 + 0.0222 x2*x3 + 0.0567 x2*x4 + 0.02831 x3*x4(3)

**Figure 20:**
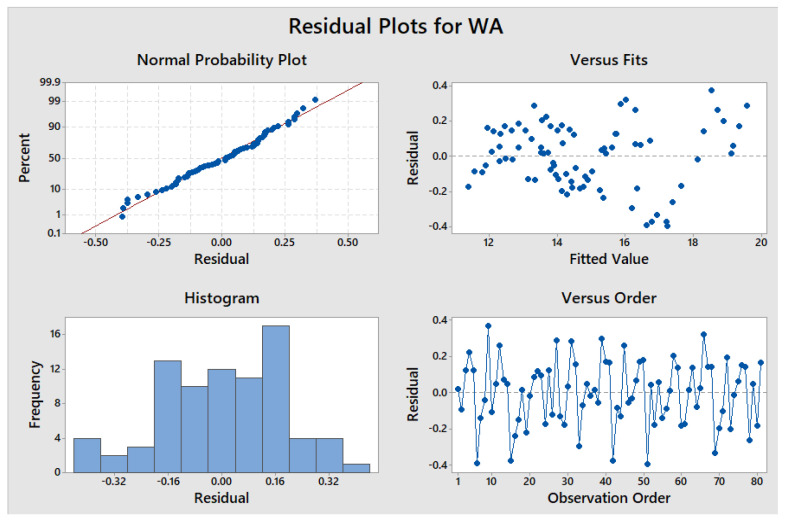
Residual plots of water absorption.

The x1, x2, x3, and x4 represent the parameters, i.e., length of PSF, the proportion of PSF, SCBA, and MD, respectively. The residual plots of WA are shown in Figure 20, where the independent variable is on the horizontal axis and the residuals are displayed on the vertical axis. The R^2^ value of 99.27% was obtained through statistical analysis.

#### 3.3.2. Model Equation: Linear Shrinkage versus x1, x2, x3, x4

The association between parameters and LS was developed using regression analysis and its result is shown in Equation (4), a model equation to determine LS of unfired soil block admixed with the varied proportion of PSF, MD, and BA:

LS = 1.832 + 0.00059 x1 − 1.493 x2 − 0.0097 x3 − 0.00367 x4 − 0.000005 x1*x1 + 0.5278 x2*x2 + 0.000089 x3*x3 + 0.000044 x4*x4 − 0.002278 x1*x2 + 0.000000 x1*x3 + 0.000022 x1*x4 + 0.00056 x2*x3 + 0.00056 x2*x4 − 0.000022 x3*x4(4)

**Figure 21:**
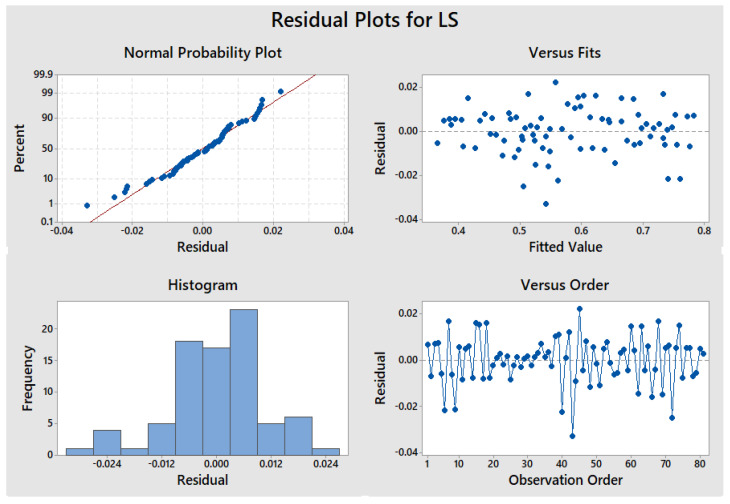
Residual plots of linear shrinkage.

The x1, x2, x3, and x4 represent the parameters, i.e., length of PSF and the proportion of PSF, SCBA, and MD respectively. The residual plots of LS are shown in Figure 21, where the independent variable is on the horizontal axis and the residuals are displayed on the vertical axis. The R^2^ value of 99.21% was obtained through statistical analysis.

### 3.4. Optimization

A search-based optimization technique called a genetic algorithm (GA) is based on the concepts of natural selection and genetics. It is frequently employed in machine learning, research, and the solution of optimization problems. In this study, the genetic algorithm (GA), an inbuilt tool in MATLAB V 17 was applied for global optimization. The values of the response factor, i.e., WA, and the parameters at optimized conditions are shown in Table 5. From this table, it can be observed that the optimum value of WA, i.e., 11.3% can be achieved by the unfired soil block admixed with 105 mm length and 0.8% PSF, 7.5% BA, and 30% MD. These optimum conditions of WA resulted by applying GA on Equation (3).

Similarly, the values of the response factor, i.e., LS, and the parameters at optimized conditions are shown in Table 6. From this table, it can be observed that the optimum value of linear shrinkage, i.e., 0.37% can be achieved by the unfired soil block admixed with 125 mm length and 1.2% PSF, 12.5% BA, and 25% MD. These optimum conditions of LS resulted by applying GA on Equation (4).

## 4. Conclusions

The study’s main goal was to determine the applicability of unfired soil blocks admixed with marble dust, paddy straw fiber, and bagasse ash from the perspective of physical parameters. The design of experiments was planned and various tests were conducted on the 81 mix designs of the prepared unfired admixed soil blocks as per the standard codes to determine the physical properties of the block. Linear shrinkage and water absorption were evaluated to estimate the physical attributes. Linear regression analysis was performed on the results and the optimized values were calculated from the modeling equations using the optimization technique. The various conclusions that were drawn from the tests are discussed below.

While estimating the water absorption (WA), it was found that WA rises with a rise in BA for the fixed amount of MD and PSF. However, the WA of the soil block was observed to be declining with a rise in MD up to 30% for the fixed amount of BA and PSF. Also, the WA tends to decline with a rise in the length of PSF but rises with rising in the content of PSF at a fixed amount of BA and MD.

At optimized conditions, the optimum value of WA for soil block was estimated with 105 mm length and 0.8% PSF, 7.5% BA, and 30% MD, i.e., 11.3%, which is less than the critical value of 20% as per standard codes. This results in a 43.5% lower value of water absorption of the block from its critical value.

The PSF content and length have a great effect on the linear shrinkage (LS) of the block, as it drastically reduced with a rise in content and length of the PSF at a fixed amount of MD and BA. The LS of the soil block was observed to be declining with a rise in BA for the fixed amount of MD and PSF.

The optimization made it evident that the optimum value of LS was observed for the soil block with 125 mm length and 1.2% PSF, 12.5% BA, and 25% MD, i.e., 0.37%, which is much less than the critical value of 3%. This implies that the addition of PSF, MD, and BA reduces the LS of the block.

The outcomes show that the recommended technique is exceptionally effective to enhance the physical attributes of unfired admixed soil blocks, as well as an environment-friendly solution to the fired bricks.

## 5. Scope of Future Work

Further research can be done on the impact of other types of natural and artificial fibers on the properties of soil blocks admixed with marble dust and bagasse ash. This could be coir, banana fiber, plastic fibers extruded from plastic bags, disposable plastic products, etc. This would greatly boost the usage of plastic trash and natural waste fibers in the building sector.

Further research can be performed on the impact of other types of binders on the properties of unfired admixed soil blocks reinforced with PSF.

## Figures and Tables

**Table 1:** Clayey soil properties.

Soil Properties	Specific Gravity	Optimum Moisture Content (%)	Liquid Limit (%)	Plasticity Index (%)	Plastic Limit (%)	Maximum Dry Density (kg/m^3^)	Unified Soil Classification System
Value	2.66	19	42.3	19.1	23.2	1670	CI

**Table 2:** Chemical characteristics of marble dust.

Constituents	SiO_2_	Al_2_O_3_	Fe_2_O_3_	CaO	MgO	SO_3_	Na_2_O	K_2_O	P_2_O_5_	Cl^-^	SrO	L.O. I
%age	0.78	0.22	0.07	54.82	0.26	0.25	0.11	0.03	0.05	0.06	0.05	43.22

**Table 3:** SCBA chemical composition.

Constituents	SiO_2_	MgO	Fe_2_O_3_	Na_2_O	K_2_O	CaO	Al_2_O_3_	SO_3_	P_2_O_5_	Other Oxides
%age	74.14	3.68	1.73	0.51	5.67	4.65	2.32	1.69	4.37	1.24

**Table 4:** Design mix using marble dust, paddy straw fiber, and bagasse ash in soil block.

Paddy Straw Fiber Length (mm) x1	Paddy Straw Fiber Content (%) x2	Bagasse Ash (%) x3	Marble Dust (%) x4
75	0.8	7.5	25
100	1	10	30
125	1.2	12.5	35

**Table 5:** Optimized value of the WA for various parameters at optimized conditions.

Response Factor/Parameter	PSF Length (mm)	PSF Content (%)	SCBA Content (%)	MD Content (%)	Optimum Value of Response Factor
	x1	x2	x3	x4	
Water Absorption	105	0.8	7.5	30	11.3

**Table 6:** Optimized value of the linear shrinkage for various parameters at optimized conditions.

Response Factor/Parameter	PSF Length (mm)	PSF Content (%)	SCBA Content (%)	MD Content (%)	Optimum Value of Response Factor
	x1	x2	x3	x4	
Linear Shrinkage	125	1.2	12.5	25	0.37

## Data Availability

No data were used to support this study.
